# Pharmacological Interventions for Alcohol Withdrawal Syndrome Among Hospitalized Adults: A Multicenter Cohort Study

**DOI:** 10.1007/s11606-025-09817-8

**Published:** 2025-08-28

**Authors:** Brody A. Erfle, Tessa L. Steel, Anica C. Law, David A. Kaufman, Kelsey Hills-Dunlap, Majid Afshar, Allan J. Walkey, Kirsten E. Austad, Mari-Lynn Drainoni, Nicholas A. Bosch

**Affiliations:** 1 Boston University Chobanian & Avedisian School of Medicine, Boston, MA USA; 2 Harborview Medical Center, Division of Pulmonary, Critical Care and Sleep Medicine, Department of Medicine, University of Washington, Seattle, WA USA; 3 The Pulmonary Center, Boston University Chobanian & Avedisian School of Medicine, 72 East Concord St., R-304, Boston, MA 02118 USA; 4 Division of Pulmonary, Critical Care and Sleep Medicine, NYU School of Medicine, New York, NY USA; 5 Division of Pulmonary Sciences and Critical Care Medicine, Department of Medicine, University of Colorado Anschutz Medical Campus, Aurora, CO USA; 6 Division of Pulmonary and Critical Care Medicine, Department of Medicine, University of Wisconsin, Madison, WI USA; 7 Division of Health Systems Science, Department of Medicine, UMass Chan Medical School, Worcester, MA USA; 8 Division of Pulmonary, Allergy and Critical Care, Department of Medicine, UMass Chan Medical School, Worcester, MA USA; 9 Evans Center for Implementation and Improvement Sciences, Department of Medicine, Boston University Chobanian & Avedisian School of Medicine, Boston, MA USA; 10 Department of Family Medicine, Boston University Chobanian & Avedisian School of Medicine, Boston, MA USA; 11 Section of Infectious Diseases, Department of Medicine, Boston Medical Center and Boston University Chobanian & Avedisian School of Medicine, Boston, MA USA; 12 Department of Health Law, Policy & Management, Boston University School of Public Health, Boston, MA USA

## Abstract

**Background:**

Alcohol Withdrawal Syndrome occurs in 1 in 43 hospitalized patients and is associated with increased morbidity. Pharmacotherapy for patients hospitalized with alcohol withdrawal syndrome are incompletely described. Understanding current practice patterns is essential to improving outcomes and addressing variability in care.

**Objective:**

To identify alcohol withdrawal syndrome treatment practice patterns across United States hospitals.

**Design:**

Multicenter retrospective cohort study with data collected between quarter 1 of 2016 and quarter 3 of 2022 using Premier Incorporated Artificial Intelligence Healthcare Database (~ 25% of United States hospital admissions; excludes emergency room care).

**Participants:**

A total of 245,271 adult inpatients with a primary diagnosis of alcohol withdrawal syndrome.

**Main Measures:**

The outcomes were alcohol withdrawal syndrome medication treatment patterns, including initial treatment regimens, dosage trends, and factors associated with medication choice.

**Key Results:**

Among 245,271 patients with alcohol withdrawal syndrome, 226,816 [92.5%] received benzodiazepines, 28,123 (11.5%) phenobarbital, and 113,746 (46.4%) other AWS agents within the first five days of admission. The most common initial treatment regimens were benzodiazepines alone (56.0%), benzodiazepines and other agents together (22.2%), and benzodiazepines and phenobarbital together (3.8%). Median daily benzodiazepine dose (diazepam equivalents) peaked on hospital day 2 (46.2 [interquartile range 23.1–84.2] mg), whereas median daily phenobarbital dose peaked on hospital day 1 (260 [120–520] mg). Type of medication used was associated with level of care at admission, hospital facility, and United States geographical region. In adjusted analyses, the admission hospital was associated with phenobarbital use (median odds ratio 4.21 [95% confidence interval 4.03–4.40]), benzodiazepine use (median odds ratio 3.09 [2.96–3.23]) and other agents (median odds ratio 1.65 [1.62–1.67]).

**Conclusions:**

Alcohol withdrawal syndrome treatment varied by level of care at admission and hospital, highlighting significant variability in practice patterns. These findings inform future trials comparing the effectiveness of different medication regimens.

**Supplementary Information:**

The online version contains supplementary material available at 10.1007/s11606-025-09817-8.

## INTRODUCTION

Alcohol Withdrawal Syndrome (AWS) – defined as central nervous system hyperstimulation resulting from the abrupt cessation of heavy, prolonged alcohol intake – occurs in approximately 700,000 hospitalized patients per year in the United States (US)^1^ and is associated with increased morbidity and mortality.^2,3^ Benzodiazepines have long been the first-line treatment for AWS.^4–6^ However, phenobarbital has emerged as a potentially viable alternative, particularly to treat severe AWS as a single, front-loaded dose. Potential equipoise between benzodiazepines and phenobarbital for the treatment of AWS in emergency room and hospitalized patients is supported by data from observational studies,^7,8^ small single-center clinical trials,^9,10^ and subsequent systematic reviews and meta-analyses^11,12^ suggesting that both treatments are effective and have comparable safety profiles. The limited data regarding phenobarbital have also contributed to contrasting guidelines regarding using phenobarbital.^5,13^ In the setting of emerging, but still limited effectiveness data, little is known about current benzodiazepine and phenobarbital practice patterns and the degree to which practice varies in the treatment of AWS in inpatient settings. Additionally, reports of other agents for AWS – propofol,^14^ ketamine,^15^ antipsychotics,^16^ alpha-2 agonists,^17,18^ and anticonvulsants^19–21^ – are increasing despite limited evidence of effectiveness. Moreover, the manner in which these other agents are being used (e.g., adjunctive, monotherapy, up-front, rescue) in the context of benzodiazepine use is unclear. Thus, there is a pressing need to benchmark current multi-center AWS medication practice patterns to identify potential areas of equipoise and usual care practices to inform future large-scale comparative effectiveness clinical trials focused on optimizing outcomes for hospitalized patients experiencing AWS.

In this study, we characterized current pharmacological practice patterns for hospitalized patients with AWS across a diverse sample of patients admitted to US hospitals.

## METHODS

### Data Source and Cohort Design:

We used the Premier Inc. Artificial Intelligence (PINC AI) Healthcare Database to generate a retrospective cohort of hospitalized patients with AWS. PINC AI includes all-payor information for inpatients discharged from 1,113 hospitals (approximately 25% of US hospitalizations); characteristics of hospitals in PINC AI are similar to those in the American Hospital Association Database.^22^ PINC AI includes patient-level demographics, International Classification of Diseases, Tenth Revision (ICD-10) codes, and daily itemized hospital charge codes that allow for identification of medications and equipment used during hospitalizations. Itemized charge codes are medication and formulation specific (e.g., phenobarbital amp 130 mg/ml; 1 ml vial) and are documented each time a medication was used allowing approximation of total daily dose. Less than 0.01% of all variable fields in PINC AI are missing data. Any preliminarily missing data received by Premier Inc. from included hospitals is returned for correction prior to final release.^23,24^ The data extract used for this study did not include emergency room or other outpatient encounters; thus, it was not possible to determine if observed medication use represented new use or continuation of emergency room or other outpatient therapies.

We included adult patients (> 18 years old) admitted between quarter 1 of 2016 to quarter 3 of 2022 with a primary admission diagnosis of alcohol withdrawal, as defined by ICD-10 codes (eTable 1). For patients with multiple admissions meeting inclusion criteria, we selected one admission at random to include in analyses.

### Outcome

We evaluated practice outcomes for three categories of medications: benzodiazepines (midazolam, lorazepam, diazepam, chlordiazepoxide), phenobarbital, and other agents potentially used as adjuncts to AWS treatment (haloperidol, carbamazepine, valproic acid, gabapentin, clonidine, dexmedetomidine, propofol, and ketamine).^25^ We separated phenobarbital from other agents given guidelines supporting its use as an alternative to benzodiazepines, particularly for severe AWS.^5,26–28^ Medication use was identified using charge codes granular to the calendar day. For each patient, we identified use of each medication on hospital days 1–5, the time period during which patients typically experience AWS.^29^ For benzodiazepines and phenobarbital, we calculated median daily dose by multiplying the formulary dose by the number of instances each medication was charged to the patient on a given calendar day. We then identified the median value (interquartile range [IQR]) of each patient’s cumulative receipt of medication on each of days 1–5 of the hospitalization (eTable 1). To quantify the combined dose of different benzodiazepines, we converted benzodiazepines to diazepam equivalents (10 mg diazepam equal to 33.3 mg chlordiazepoxide, 1.3 mg lorazepam, and 2.7 mg IV midazolam).^30^

### Covariables

We examined associations between hospital- and patient-level factors (eTable 1) and AWS practice patterns in hierarchical models. Patient-level factors included demographics, comorbidities present on admission,^31^ and level of care on admission (defined as the highest level of care received on day 1 of hospitalization: Intensive Care Unit [ICU] receiving invasive mechanical ventilation [IMV] > ICU not receiving IMV > intermediate care unit > general wards). We separated patients in the ICU to those who were receiving and not receiving IMV on day 1 given medications used to treat AWS may also be given for analgosedation during IMV. Hospital-level factors included geographical region,^32^ urban or rural setting, bed count, teaching status, safety net status, and the hospital facility.

### Analysis

We used descriptive statistics (counts [percentage] and median [IQR]) to summarize covariables stratified by level of care at admission, hospital day, hospital facility, presence of delirium tremens and level of care at admission, and case volume and level of care at admission to provide insight regarding differential patterns of medication use across treatment locations, severity of AWS, and time. We used alluvial flow diagrams^33^ to describe changes in AWS treatment regimens in individual patients from day 1 to day 5 of hospitalization. For each category of medication (benzodiazepines, phenobarbital, other agents), we used hierarchical logistic regression models including covariables as fixed effects and hospital site as a random intercept term to identify factors associated with medication use in the first 5 days of hospitalization. Associations were estimated as adjusted odds ratios (aORs) and 95% confidence intervals (CIs). We also estimated the median odds ratio, a measure of variation attributable to the hospital facility after accounting for fixed effects.^34^ We used bootstrapping to identify 95% confidence intervals for the median odds ratio. This study was deemed not human subjects research by the Boston University Institutional Review Board (#H-41795).

### Role of the Funding Source

This study was supported by the National Institutes of Health (NIH) National Center for Advancing Translational Sciences grant numbers 1KL2TR001411 and 1UL1TR001430 and the Boston University Medical Student Summer Research Program. The NIH and Boston University had no role in the design, conduct, analyses, manuscript or decision to submit the manuscript for publication. This study's contents are solely the responsibility of the authors and do not represent the official views of the NIH or Boston University.

## RESULTS

A total of 753,074 encounters included any diagnosis of AWS, of which 354,202 had a primary diagnosis of AWS. After selecting a random encounter for patients with more than one, the final study sample included 245,271 adult patients admitted to 1,086 hospitals (eFigure 1); 163,713 patients (66.7%) were admitted to general wards, 48,997 (20.0%) to intermediate care units, 27,378 (11.2%) to ICUs not receiving IMV, and 5,183 (2.1%) to ICUs receiving IMV on hospital day 1 (Table 1). The median age was 48 (IQR 38–57); patients were predominantly male (n = 180,364 [73.5%]) and white race (n = 191,741 [78.2%]). Common patient comorbidities included fluid and electrolyte disorders (n = 119,446 [48.7%]), psychosis (n = 103,227 [42.1%]), and liver disease (n = 74,456 [30.4%]); 23.1% (n = 56,582) of patients had a diagnosis of delirium tremens (Table 1). The median (IQR) length of hospital stay was 3 days (2–5 days) and 705 patients (0.3%) died during hospitalization.

**Table 1:** Characteristics of Patients Admitted for Alcohol Withdrawal Syndrome, Stratified by Level of Care

**Level of Care**	ICU and receiving IMV (n = 5,183)	ICU and not receiving IMV (n = 27,378)	Intermediate Care Unit (n = 48,997)	General Wards (n = 163,713)	Overall (n = 245,271)
**Patient Characteristics**					
Age in years, median (IQR)	50 (40, 59)	48 (38, 57)	49 (38, 58)	48 (38, 57)	48 (38, 57)
Length of stay in days, median (IQR)	9 (5, 14)	4 (2, 6)	3 (2, 5)	3 (2, 5)	3 (2, 5)
Mortality, No. (%)	215 (4.1)	56 (0.2)	152 (0.3)	281 (0.2)	704 (0.3)
Race, No. (%)					
Asian	35 (0.7)	183 (0.7)	325 (0.7)	1478 (0.9)	2021 (0.8)
Black	462 (8.9)	2061 (7.5)	4060 (8.3)	16001 (9.8)	22584 (9.2)
Other	491 (9.5)	2037 (7.4)	4245 (8.7)	16162 (9.9)	22935 (9.4)
Unreported	172 (3.3)	601 (2.2)	1129 (2.3)	4088 (2.5)	5990 (2.4)
White	4023 (77.6)	22,496 (82.2)	39,238 (80.1)	125,984 (77.0)	191,741 (78.2)
Sex, No. (%)					
Female	809 (15.6)	5970 (21.8)	12,259 (25.0)	45,782 (28.0)	64,820 (26.4)
Male	4374 (84.4)	21,387 (78.1)	36,734 (75.0)	117,869 (72.0)	180,364 (73.5)
Unreported	0 (0.0)	21 (0.1)	4 (0.0)	62 (0.0)	87 (0.0)
Hispanic Ethnicity, No. (%)					
No	182,709 (74.5)	121,278 (74.1)	36,050 (73.6)	21,599 (78.9)	3782 (73.0)
Yes	18,789 (7.7)	12,044 (7.4)	4405 (9.0)	1898 (6.9)	442 (8.5)
Unknown	43,773 (17.8)	30,391 (18.6)	8542 (17.4)	3881 (14.2)	959 (18.5)
Marital status, No. (%)					
Married	55,396 (22.6)	36,103 (22.1)	11,582 (23.6)	6368 (23.3)	1343 (25.9)
Other	24,998 (10.2)	17,393 (10.6)	4313 (8.8)	2690 (9.8)	602 (11.6)
Single	162,949 (66.4)	108,922 (66.5)	32,749 (66.8)	18,090 (66.1)	3188 (61.5)
Unknown	1928 (0.8)	1295 (0.8)	353 (0.7)	230 (0.8)	50 (1.0)
Insurance Type, No. (%)					
Medicare	41,715 (17.0)	27,778 (17.0)	8622 (17.6)	4239 (15.5)	1076 (20.8)
Medicaid	92,626 (37.8)	62,930 (38.4)	17,944 (36.6)	9797 (35.8)	1955 (37.7)
Commercial	64,035 (26.1)	44,296 (27.1)	11,643 (23.8)	6928 (25.3)	1168 (22.5)
Other	46,895 (19.1)	28,709 (17.5)	10,788 (22.0)	6414 (23.4)	984 (19.0)
Major surgery on hospital day 1, No. (%)	235 (4.5)	193 (0.7)	281 (0.6)	668 (0.4)	1377 (0.6)
Delirium tremens diagnosis, No. (%)	3323 (64.1)	13,075 (47.8)	13,182 (26.9)	27,002 (16.5)	56,582 (23.1)
Gagne Comorbidities Present On Admission*,^31,54^ No. (%)					
Metastatic cancer	13 (0.3)	44 (0.2)	84 (0.2)	224 (0.1)	365 (0.1)
Congestive heart failure	645 (12.4)	1534 (5.6)	2646 (5.4)	6441 (3.9)	11,266 (4.6)
Dementia	169 (3.3)	469 (1.7)	672 (1.4)	1935 (1.2)	3245 (1.3)
Renal failure§	219 (4.2)	705 (2.6)	1180 (2.4)	3182 (1.9)	5286 (2.2)
Weight loss	227 (4.4)	1054 (3.8)	1415 (2.9)	3927 (2.4)	6623 (2.7)
Hemiplegia	65 (1.3)	110 (0.4)	152 (0.3)	342 (0.2)	669 (0.3)
Any tumor†	53 (1.0)	179 (0.7)	365 (0.7)	1114 (0.7)	1711 (0.7)
Cardiac arrhythmias	1095 (21.1)	4740 (17.3)	8363 (17.1)	19,058 (11.6)	33,256 (13.6)
Chronic pulmonary disease	929 (17.9)	3981 (14.5)	7269 (14.8)	24,175 (14.8)	36,354 (14.8)
Coagulopathy	1596 (30.8)	7633 (27.9)	11,035 (22.5)	26,232 (16.0)	46,496 (19.0)
Complicated diabetes	305 (5.9)	1097 (4.0)	2209 (4.5)	5968 (3.6)	9579 (3.9)
Deficiency anemias‡	1242 (24.0)	4274 (15.6)	6773 (13.8)	17,315 (10.6)	29,604 (12.1)
Fluid and electrolyte disorders	3678 (71.0)	18,034 (65.9)	29,564 (60.3)	68,170 (41.6)	119,446 (48.7)
Liver disease	1973 (38.1)	10,490 (38.3)	17,199 (35.1)	44,794 (27.4)	74,456 (30.4)
Peripheral vascular disorder	147 (2.8)	382 (1.4)	712 (1.5)	1999 (1.2)	3240 (1.3)
Psychosis	1575 (30.4)	9913 (36.2)	18,212 (37.2)	73,527 (44.9)	103,227 (42.1)
Pulmonary circulation disorders	102 (2.0)	203 (0.7)	334 (0.7)	844 (0.5)	1483 (0.6)
Hypertension	546 (10.5)	1495 (5.5)	2682 (5.5)	6649 (4.1)	11,372 (4.6)
Present on Admission Unweighted Gagne Comorbidity Score, median (IQR)	3 (2, 4)	2 (1, 3)	2 (1, 3)	2 (1, 3)	2 (1, 3)
**Hospital Factors**					
Urban hospital, No. (%)	4519 (87.2)	22,933 (83.8)	44,891 (91.6)	147,585 (90.1)	219,928 (89.7)
Safety net hospital, No. (%)	7495 (27.4)	1614 (31.1)	13,427 (27.4)	50,101 (30.6)	72,637 (29.6)
Teaching hospital, No. (%)	11,283 (41.2)	2714 (52.4)	20,662 (42.2)	83,808 (51.2)	118,467 (48.3)
Hospital Bed Count, No. (%)					
0–99	3511 (12.8)	348 (6.7)	2311 (4.7)	10,471 (6.4)	16,641 (6.8)
100–199	5643 (20.6)	772 (14.9)	9355 (19.1)	21,590 (13.2)	37,360 (15.2)
200–299	4540 (16.6)	893 (17.2)	8922 (18.2)	30,639 (18.7)	44,994 (18.3)
300–399	4386 (16.0)	822 (15.9)	8068 (16.5)	28,603 (17.5)	41,879 (17.1)
400–499	2998 (11.0)	662 (12.8)	5454 (11.1)	20,334 (12.4)	29,448 (12.0)
500 +	6300 (23.0)	1686 (32.5)	14,887 (30.4)	52,076 (31.8)	74,949 (30.6)
United States Census Division, No. (%)					
East North Central	914 (17.6)	5551 (20.3)	9337 (19.1)	30,582 (18.7)	46,384 (18.9)
East South Central	259 (5.0)	1829 (6.7)	1428 (2.9)	7715 (4.7)	11,231 (4.6)
Middle Atlantic	642 (12.4)	2778 (10.1)	5184 (10.6)	31,735 (19.4)	40,339 (16.4)
Mountain	465 (9.0)	1713 (6.3)	6939 (14.2)	10,544 (6.4)	19,661 (8.0)
New England	277 (5.3)	558 (2.0)	1655 (3.4)	8307 (5.1)	10,797 (4.4)
Pacific	610 (11.8)	3131 (11.4)	5291 (10.8)	15,989 (9.8)	25,021 (10.2)
South Atlantic	1180 (22.8)	5693 (20.8)	13,905 (28.4)	35,512 (21.7)	56,290 (23.0)
West North Central	389 (7.5)	3123 (11.4)	2135 (4.4)	10,613 (6.5)	16,260 (6.6)
West South Central	447 (8.6)	3002 (11.0)	3123 (6.4)	12,716 (7.8)	19,288 (7.9)

ICU: intensive care unit; IMV: intensive mechanical ventilation; IQR: interquartile range

^*^Gagne comorbidities refers to validated measures of comorbidity severity based on International Classification of Diseases diagnosis codes that were developed by Gagne et al.^55^ and adapted by Sun et al.^54^

^†^Any tumor refers to International Classification of Diseases, Tenth Revision Codes for hematologic and solid malignancies that are not explicitly metastatic

^‡^Deficiency anemias refer to International Classification of Diseases, Tenth Revision Codes for nutritional anemias including iron deficiency anemia, vitamin B12 deficiency anemia, and folate deficiency anemia

^§^Renal failure refers to International Classification of Diseases, Tenth Revision Codes for chronic kidney disease or unspecified kidney failure

### Variation in AWS Medication use by Level of Care at Admission and Hospital

**Figure 1:**
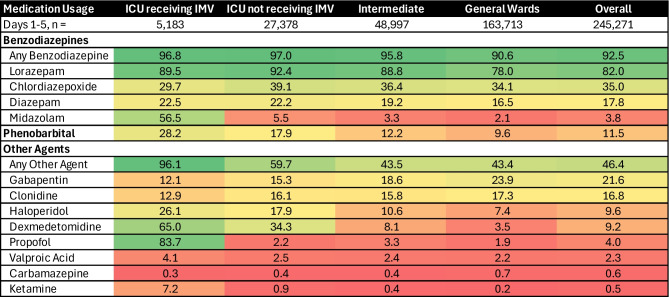
Heatmap of AWS medication use, overall and stratified by level of care at admission. Dark green corresponds to medications with the highest use. Dark red corresponds to medications with the lowest use. The column headings correspond to patients’ level of care on the first day of hospitalization (i.e., at admission). Percentages represent medication use over the first 5 days of admission. Thus, for a hypothetical patient admitted to the General Wards on hospital day 1 who was transferred to the intensive care unit on hospital day 2 and received propofol on hospital day 2 in the intensive care unit, propofol use would be included in the column for General Wards because that was the level of care at the time of admission.

Most patients received a benzodiazepine within the first five days of admission (n = 226,816 [92.5%]). The most frequently administered benzodiazepines were lorazepam (n = 201,106 [82.0%]), chlordiazepoxide (n = 85,808 [35.0%]), diazepam (n = 43,688 [17.8%]), and midazolam (n = 9,418 [3.8%]). Benzodiazepines were used at similar rates across levels of care at admission except for midazolam (Fig. 1), which was more likely to be used in patients admitted to ICUs who were receiving IMV (n = 2,928 [56.5%]).

Phenobarbital was used in 11.5% (n = 28,123) of patients and was more common in patients admitted to higher levels of care (Fig. 1). Less than half of all cohort patients (n = 113,746 [46.4%]) received an agent other than phenobarbital or benzodiazepines, but use of medications for AWS varied by admission location (e.g., 96.1% [n = 4,980] of patients admitted to ICUs who were receiving IMV vs. 43.4% [n = 71,122] of patients admitted to general wards) and by the specific agent (e.g., 21.6% [n = 53,085] received gabapentin vs. 0.5% [n = 1,209] received ketamine). Patients with delirium tremens were more likely to be cared for in higher acuity settings and were more likely to receive phenobarbital and other adjuncts relative to patients without delirium tremens (eFigure 2). There were few differences in practice between patients admitted to low case volume hospitals and those admitted to high case volume hospitals (eFigure 3).

**Figure 2:**
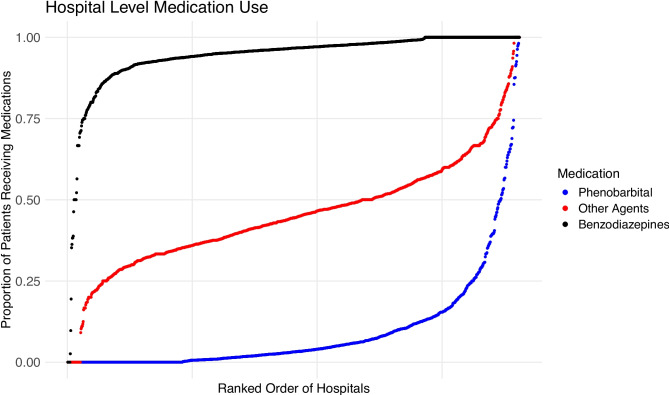
Proportion of patients at the hospital level receiving each medication class. Each data point corresponds to an individual hospital. Hospitals are rank-ordered along the x-axis by their prevalence of medication use; hospitals are reordered for each medication.

The median (IQR) hospital-level use of benzodiazepines, phenobarbital, and other agents was 96.7% (93.7–98.9%), 3.2% (0–10.5%), and 44.4% (35.0–54.2%), respectively (Fig. 2).

### AWS Treatment Regimens During Hospitalization

**Figure 3:**
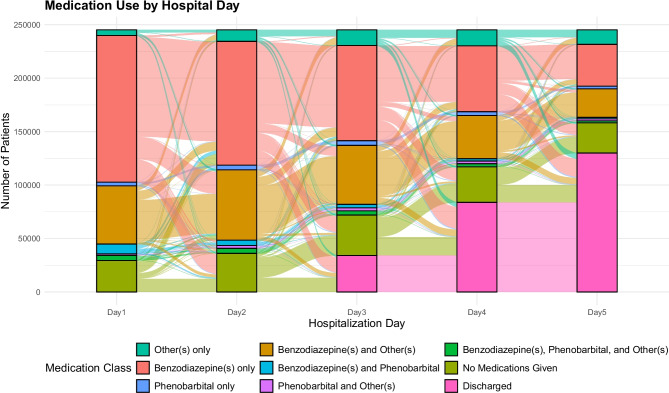
Alluvial flow diagram of medication classes received during hospital days 1 through 5. Each column corresponds to a given hospital day; colors correspond to the number of patients on each patient day receiving a given combination of medications. Lines connecting columns correspond to changes in medications by day.

On the first day of hospitalization, the most frequent practices were benzodiazepines alone (n = 137,300 [56.0%]), benzodiazepines and other agents together (n = 54,456 [22.2%]), and benzodiazepines and phenobarbital together (n = 9,200 [3.8%]) (Fig. 3). Other agent use (in combination or alone) increased from hospital day 1 (n = 65,959 [26.9%]) to hospital day 3 (n = 76,688 [36.3%]) whereas use of benzodiazepines alone decreased (day 3: n = 89,024 [42.2%]). Phenobarbital was rarely the only AWS medication prescribed on a given calendar day (< 2.1% of all calendar days). By hospital day 5, 64.5% (n = 158,236) of all cohort patients had either been discharged or taken off all measured AWS treatments (Fig. 3). AWS practices on the wards and intermediate care units (non-ICU) were generally similar to overall treatment regimen patterns (eFigures 4 and 5), whereas patients admitted to ICUs experienced unique patterns of treatment, especially with IMV. Compared to non-ICU patients, patients admitted to ICUs with IMV were less likely to receive benzodiazepine monotherapy on day 1 (15.1% [n = 783] versus 58.0% [n = 123,305]) and more likely to receive combination treatment with benzodiazepines and other agents (56.4% [n = 2,924] versus 20.2% [n = 42,912]) or all three categories of medications (13.0% [n = 676] versus 1.3% [n = 2,694]). These differences were present but less pronounced among patients admitted to ICUs without IMV. Patients admitted to ICUs more frequently continued on AWS treatments through hospital day 5 (48.0% [n = 15,652] versus 33.6% [n = 71,383]) (eFigures 6 and 7).

### Factors Associated with AWS Medication Use

Higher levels of care were associated with increased odds of benzodiazepine, phenobarbital, and other agent use (eTables 2 and 3). Compared to patients admitted to general wards, patients admitted to ICUs receiving IMV had 6.72 (95% CI 6.19–7.29) and 61.45 (95% CI 51.13–73.85) times greater adjusted odds of phenobarbital and other agent use, respectively (no difference in benzodiazepine use); patients admitted to ICUs not receiving IMV had 2.11 (95% CI 1.87–2.37), 3.87 (95% CI 3.68–4.07), and 2.29 (95% CI 2.22–2.36) times greater adjusted odds of benzodiazepine, phenobarbital, and other agent use, respectively; and patients admitted to intermediate care units had 1.93 (95% CI 1.14–1.21) and 1.49 (95% CI 1.42–1.56) times greater odds of benzodiazepine and phenobarbital use, respectively (no difference in use of other agents).

Each 1-year increase in age was associated with lower odds of benzodiazepine (aOR 0.91 [95% CI 0.89–0.93]) and phenobarbital use (aOR 0.85 [95% CI 0.84–0.87]), but higher odds of other agent use (aOR 1.04 [95% CI 1.03–1.05]). Teaching hospitals were associated with increased odds of phenobarbital use (aOR 1.48 [95% CI 1.13–1.95]), and decreased odds of other agent use (aOR 0.88 [95% CI 0.79–0.97]).

Practices were variable by geographic region, especially phenobarbital, which was more likely in New England (aOR compared to reference East North Central 6.33 [95% CI 3.12–12.84]) (eFigure 8). After accounting for covariables, the hospital facility itself was associated with phenobarbital use (median odds ratio 4.21 [95% CI 4.03–4.40]) and benzodiazepine use (median odds ratio 3.09 [2.96–3.23]) and associated with use of other agents (median odds ratio 1.65 [1.62–1.67]).

### Benzodiazepine and Phenobarbital Daily Dosing

**Figure 4:**
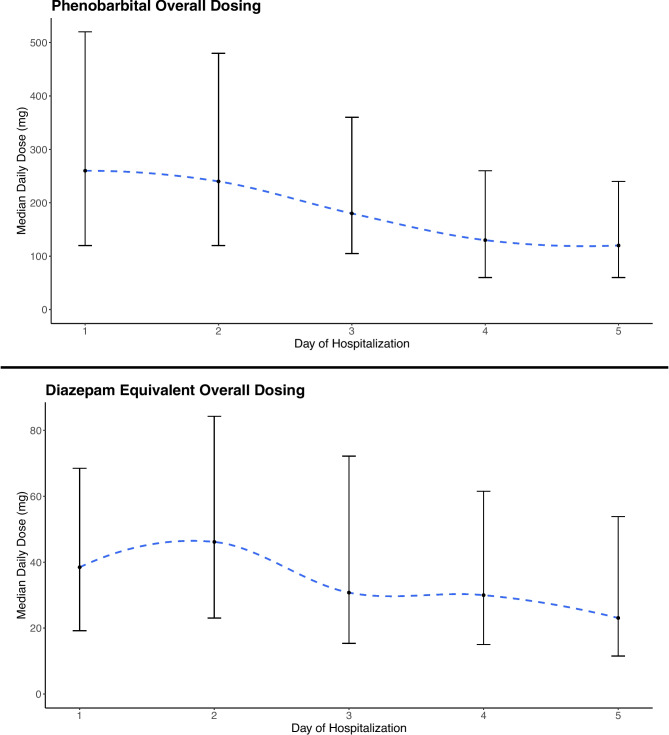
Phenobarbital and diazepam equivalent daily dose. Median (point) total daily dose and interquartile range (whiskers) of phenobarbital (top panel) and diazepam equivalents (bottom panel) over time. Dosing information for a given hospital day was collected from patients who received at least one dose of the relevant medication (phenobarbital or a benzodiazepine) on that specific day.

The overall median (IQR) daily benzodiazepine dose (diazepam equivalents) on hospital day 1 was 38.5 mg (19.2–68.5 mg). Day 1 median daily benzodiazepine doses were highest among patients in the ICU receiving IMV (92.3 [40.8–353.0] mg) and lowest among patients on the general wards (30.7 [15.4–60.8] mg). Median daily benzodiazepine dose peaked on hospital day 2 (46.2 [23.1–84.2] mg) while median phenobarbital dose peaked on hospital day 1 (260 [120–520] mg) (Fig. 4).

## DISCUSSION

In this multicenter retrospective cohort study of hospitalized patients with AWS, AWS pharmacotherapy was highly variable. Although over 90% of patients received benzodiazepines, there was large variation in the use of specific benzodiazepines, phenobarbital, and other agents across levels of care, days in the hospital, hospital facilities, and geographic regions. These results provide critical data for benchmarking hospital performance and highlight the equipoise in diverse usual care practices, providing rationale for pragmatic comparative effectiveness clinical trials to evaluate which treatments are most effective for which types of patients.

Our results should be considered in the context of prior work. Benzodiazepines are the first-line treatment for AWS and are recommended by guidelines.^5,28,35^ In a clinician survey, benzodiazepine monotherapy was identified by the majority of respondents as the treatment of choice in mild to moderate AWS.^36^ We similarly identified benzodiazepine monotherapy as the most common AWS practice, although use of other agents was also common, especially in higher levels of care (potentially correlating with higher severity of AWS or other acute illness) and at later time points during hospitalization. Among benzodiazepines, long acting agents with active metabolites (e.g., diazepam; chlordiazepoxide) are favored by guidelines due to “self-tapering” properties.^2,5,37^ However, we identified that lorazepam – a benzodiazepine with an intermediate half-life, no active metabolites^38^ – was the predominant benzodiazepine used in AWS across all levels of care. Lorazepam was similarly found the be the most prescribed benzodiazepine in a national study of hospital-based AWS treatment practices in the Veteran’s Health Administration.^39^ Future studies should investigate contextual factors associated with – potentially guideline discordant – use of short- and intermediate-acting benzodiazepines.

In 2016 and 2018 surveys,^36,40^ clinicians self-reported haloperidol as the most frequently given adjunct for AWS. A 2024 study of emergency room practices identified gabapentinoids and valproic acid derivatives as the most frequently used additional agents.^41^ In contrast, both clonidine and gabapentin were more frequently used as additional agents in our study compared to haloperidol. The decreased haloperidol use observed in our study relative to prior work may suggest increasing safety concerns for anti-psychotics in hospitalized patients and the potential for decreased seizure threshold.^42–44^ Large observed variation in other agent use may also reflect discrepant recommendations regarding the use of antipsychotics and alpha-2 agonists as routine adjunctive treatments in AWS.^2,5^ Evidence supporting use of propofol, dexmedetomidine, and ketamine infusions in the management of AWS is largely anecdotal.^45,46^ Our multicenter results demonstrate that these infusions are used relatively frequently across the United States, especially among patients receiving IMV, but there is a need for more robust effectiveness data specifically for AWS to inform practice beyond sedation used for mechanical ventilation.

Our results inform the design of future clinical trials seeking to optimize AWS management for hospitalized patients. Although observational evidence suggests phenobarbital may be helpful as an adjunct or as monotherapy,^7,8,47^ there remains scant randomized controlled trial evidence of phenobarbital effectiveness.^10,48^ While few patients in our cohort received phenobarbital, prior work suggests that phenobarbital use is increasing.^8^ We found a strong association between phenobarbital use and geographical region, especially in New England. This large variation may reflect regional implementation of phenobarbital via diffusion of practice patterns. ^7,49–51^ Prior to widespread implementation, the field needs stronger evidence of effectiveness, especially comparing phenobarbital to benzodiazepines with similar pharmacokinetics (e.g., diazepam; chlordiazepoxide).

Phenobarbital dose peaked on day 1 whereas diazepam equivalents dose on day 2, a pattern that may suggest that phenobarbital is typically front-loaded (administered as a large initial dose).^25^ These results may suggest that a future clinical trial may require a factorial design to simultaneously evaluate both medication type and dosing strategies. The large observed variation in other agent usage across hospitals and among differing levels of care may make usual care study arms difficult to standardize and implement. Clinical trials with adaptive randomization or adaptive interventions may thus be best suited to study the effectiveness of AWS medications.^52^ Given the large differences in AWS treatments delivered to patients who are receiving IMV, future clinical trials should be designed to consider unique treatment needs and effects in this important patient subgroup.

Our study has limitations. PINC AI is granular to the hospital day which may miss important differences in AWS management that occur over shorter time intervals. We did not have access to pre-admission data, including emergency room data, that may influence inpatient AWS treatment decisions. Thus, it is possible that observed inpatient medication practices represent continuation of outpatient “home” medications, that treatments administered in the emergency room (e.g., monotherapy loads, rescue medications) influenced medication selection during hospitalization and initial admission triage decisions, and that observed rates of phenobarbital and of self-tapering, long-acting benzodiazepines (both of which may be given as front-loaded doses in the emergency room) may be underestimates. We were unable to accurately identify route of administration, and differences in oral, intravenous, or intramuscular medication use could not be assessed. Although models accounted for delirium tremens and potential indirect markers of AWS severity (mechanical ventilation, level of care), we did not have access to standardized measures of AWS symptom severity (e.g., revised clinical institute withdrawal assessment for alcohol scale [CIWA-Ar]);^53^ thus, the degree to which the observed variation in AWS treatments was attributable to differences in symptom severity is unclear. We could not determine the intent of observed practices and thus could not distinguish between medications being used for AWS versus other indications (e.g., sedation during IMV). However, we purposely limited the study to patients with a primary diagnosis of AWS to increase the likelihood that observed patterns represented treatment for AWS. The method used to calculate total daily dose has not been validated against gold standard chart review.

## CONCLUSION

In this multicenter cohort study of hospitalized patients with AWS, we observed substantial variation in medication choice and dosing across levels of care, days in the hospital, hospital facilities, and geographic regions, in the management of inpatient AWS. These results inform – and highlight the urgent need for – well-designed randomized clinical trials focused on optimizing and standardizing the management of AWS.

## Supplementary Information

Below is the link to the electronic supplementary material.

Supplementary file1 (DOCX 1459 KB)

## Data Availability

The Data Use Agreement Between Boston University and Premier Inc. prevent sharing of the raw data used for this study.
