# Supplementary material for: Pharmacological Interventions for Alcohol Withdrawal Syndrome Among Hospitalized Adults: A Multicenter Cohort Study
Source: J Gen Intern Med. 2025 Aug 28;41(4):1058–68. doi: 10.1007/s11606-025-09817-8 (PMC12683996; doi:10.1007/s11606-025-09817-8)
Supplement: Supplementary file 1 — Supplementary file1 (DOCX 1459 KB) [file 11606_2025_9817_MOESM1_ESM.docx]

**Pharmacological Interventions for Alcohol Withdrawal Syndrome Among Hospitalized Adults: A Multicenter Cohort Study**

**Online-only Material**

eTable 1: Study Variable Definitions

eFigure 1: Cohort Inclusion and Exclusion Criteria

eFigure 2: Heatmaps of AWS medication use stratified by presence of delirium tremens and level of care at admission. Dark green corresponds to medications with the highest use. Dark red corresponds to medications with the lowest use.

eFigure 3: Heatmaps of AWS medication use stratified by hospital case volume and level of care at admission. Dark green corresponds to medications with the highest use. Dark red corresponds to medications with the lowest use.

eFigure 4: Medication Flow Diagram for patients initially admitted to Intermediate Care

eFigure 5: Medication Flow Diagram for patients initially admitted to General Wards

eFigure 6: Medication Flow Diagram for patients initially admitted to ICUs and receiving IMV

eFigure 7: Medication Flow Diagram for patients initially admitted to ICUs and not receiving IMV eTable 2: Adjusted Odds Ratios for Benzodiazepine and Phenobarbital Administration

eTable 3: Adjusted Odds Ratios for Other Agent Administration

eFigure 8: Forest Plot of Other Agent Odds Ratios by Hospital Location

eReferences

**eTable 1: Study Variable Definitions**

| **Variable** | **Definition** |
| --- | --- |
| **Inclusion/exclusion** |  |
| Adults | DEMOGRAPHICS table AGE variable ≥18 |
| Primary diagnosis of alcohol withdrawal | PAT_ICDDIAG table ICD_CODEs (ICD-10) equal to F10.230, F10.231, F10.232, F10.239, F10.130, F10.131, F10.132, F10.139, F10.930, F10.931, F10.932, F10.939 with ICD_POA (present on admission identifier) equal to “Y” and ICD_PRI_SEC (primary diagnosis identifier) equal to “P” |
| **Medications** |  |
| Phenobarbital | PATBILL table STD_CHG_CODE matches PROD_NAME_DESC equal to "PHENOBARB " and SERV_DAY was between 1-5 |
| Lorazepam | PATBILL table STD_CHG_CODE matches PROD_NAME_DESC equal to "LORAZEPAM " and SERV_DAY was between 1-5 |
| Chlordiazepoxide | PATBILL table STD_CHG_CODE matches PROD_NAME_DESC equal to "CHLORDIAZEPOXIDE " and SERV_DAY was between 1-5 |
| Diazepam | PATBILL table STD_CHG_CODE matches PROD_NAME_DESC equal to "DIAZEPAM " and SERV_DAY was between 1-5 |
| Midazolam | PATBILL table STD_CHG_CODE matches PROD_NAME_DESC equal to "MIDAZOLAM " and SERV_DAY was between 1-5 |
| Clonidine | PATBILL table STD_CHG_CODE matches PROD_NAME_DESC equal to "CLONIDINE " and SERV_DAY was between 1-5 |
| Gabapentin | PATBILL table STD_CHG_CODE matches PROD_NAME_DESC equal to "GABAPENTIN " and SERV_DAY was between 1-5 |
| Haloperidol | PATBILL table STD_CHG_CODE matches PROD_NAME_DESC equal to "HALOPERIDOL " and SERV_DAY was between 1-5 |
| Valproic Acid | PATBILL table STD_CHG_CODE matches PROD_NAME_DESC equal to "VALPROIC ACID" or “DIVALPROEX NA” and SERV_DAY was between 1-5 |
| Dexmedetomidine | PATBILL table STD_CHG_CODE matches PROD_NAME_DESC equal to "CLONIDINE " and SERV_DAY was between 1-5 |
| Propofol | PATBILL table STD_CHG_CODE matches PROD_NAME_DESC equal to "PROPOFOL " and SERV_DAY was between 1-5 |
| Ketamine | PATBILL table STD_CHG_CODE matches PROD_NAME_DESC equal to "KETAMINE " and SERV_DAY was between 1-5 |
| Carbamazepine | PATBILL table STD_CHG_CODE matches PROD_NAME_DESC equal to "CARBAMAZEPINE " and SERV_DAY was between 1-5 |
| **Covariables** |  |
| Admission to the ICU on hospital day 1 | PATBILL table STD_CHG_CODE matches CLIN_SUM_DESC equal to "R&B ICU" and SERV_DAY was equal to 1 |
| Admission to the intermediate care unit on hospital day 1 | PATBILL table STD_CHG_CODE matches CLIN_SUM_DESC equal to "R&B STEP DOWN" and SERV_DAY was equal to 1 |
| Invasive mechanical ventilation on hospital day 1 | PATBILL table STD_CHG_CODE equal to 270270013950000, 270270057050000, 270270086600000, 270270088900000, 270270089300000, 290290093620000, 410412946560000, 410412946560001, 410412946570000, 410412946570004, 410412946570005, 410412946570007, 410412946570009, 970976946560000, 970976946570000 and SERV_DAY was equal to 1 |
| Hospital of Admission | DEMOGRAPHICS table PROV_ID variable |
| Age | DEMOGRAPHICS table AGE variable |
| Race | DEMOGRAPHICS table RACE variable |
| Ethnicity | DEMOGRAPHICS table HISPANIC_IND variable |
| Marital status | DEMOGRAPHICS table MART_STATUS variable |
| Insurance | DEMOGRAPHICS table SD_PAYOR variable divided into categories of Medicare, Medicaid, Commercial, and Other |
| Sex | DEMOGRAPHICS table GENDER variable equal to “F” for female, “M” for male, “U” for unreported |
| Hospital Setting, Urban | DEMOGRAPHICS table URBAN_RURAL variable equal to “URBAN” |
| Surgery during admission | PAT_ICDPROC table ICD_CODE equal to those in the Healthcare Cost and Utilization Project Database ^1^ labeled as major diagnostic or therapeutic and PROC_DAY equal to 1 |
| Delirium Tremens during admission | PAT_ICDDIAG table ICD_CODE equal to "F10.231", "F10.131", "F10.931" |
| Safety hospital status | Hospitals at or above the 75% for a given US Census Division in their rate of encounters where the insurance type (DEMOGRAPHIC TABLE STD_PAYOR variable) is for Medicaid or free care. |
| Teaching Hospital status, yes | PROVIDERS table TEACHING variable equal to “YES” |
| United States Division | PROVIDERS table PROV_DIVISION variable |
| Hospital Size, beds | PROVIDERS table BEDS_GRP variable |
| Gagne Comorbidity Measures present on admission | PAT_ICDDIAG and PAT_ICDPROC table ICD_CODE equal to those used in Gagne et al. and ICD_POA equal to “Y” or PROC_DAY equal to 1^2^ |
| Year of admission | DEMOGRAPHICS table ADM_MON character string in the first 4 digits |

**eFigure 1: Cohort Inclusion and Exclusion Criteria**

**
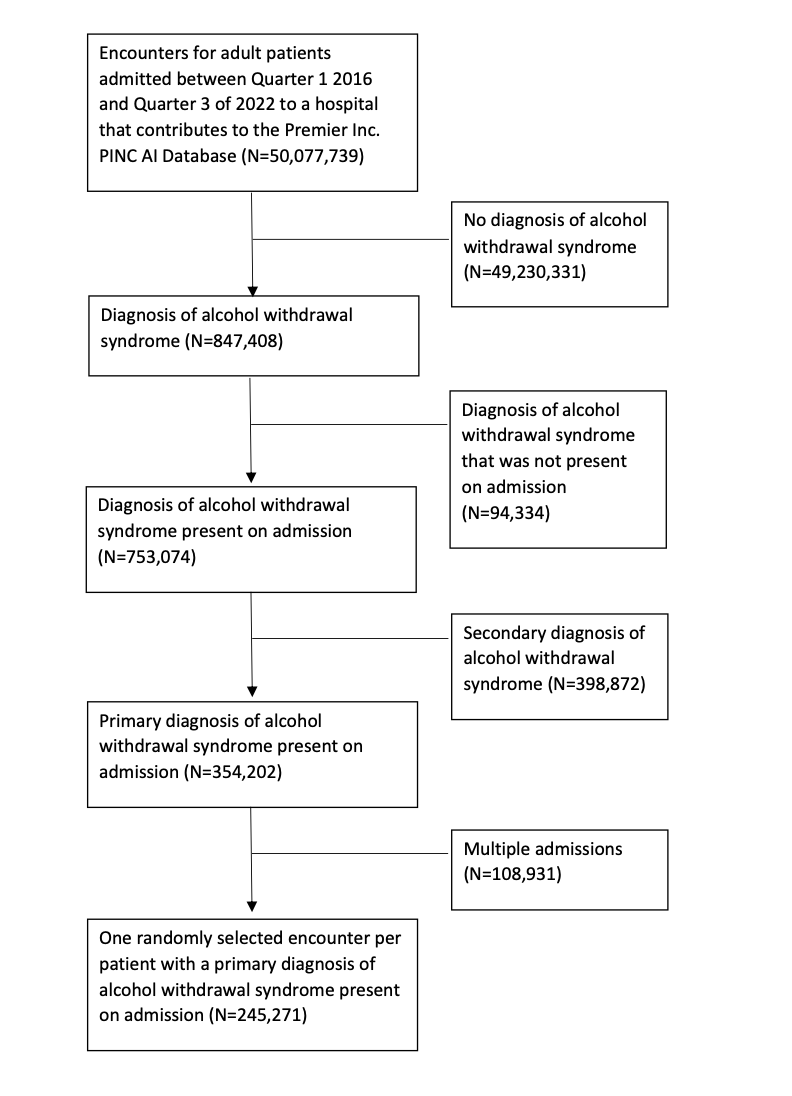
**

Flow of patient inclusion and exclusion criteria in AWS cohort formation

**eFigure 2: Heatmaps of AWS medication use stratified by presence of delirium tremens and level of care at admission**

| **Delirium Tremens Medication Usage** | **ICU receiving IMV** | **ICU not receiving IMV** | **Intermediate** | **General Wards** | **Overall** |
| --- | --- | --- | --- | --- | --- |
| Days 1-5, n = | 3,323 | 13,075 | 13,182 | 27,002 | 56,582 |
| **Benzodiazepines** |  |  |  |  |  |
| Any Benzodiazepine | 97.7 | 98.0 | 97.7 | 95.2 | 96.6 |
| Lorazepam | 92.2 | 94.6 | 93.5 | 89.7 | 91.8 |
| Chlordiazepoxide | 32.8 | 41.2 | 41.7 | 39.1 | 39.8 |
| Diazepam | 24.8 | 24.7 | 21.4 | 20.1 | 21.8 |
| Midazolam | 58.3 | 6.9 | 5.6 | 5.2 | 8.8 |
| **Phenobarbital** | 32.0 | 22.5 | 17.2 | 14.8 | 18.1 |
| **Other Agents** |  |  |  |  |  |
| Any Other Agent | 96.9 | 71.0 | 54.8 | 52.0 | 59.7 |
| Gabapentin | 12.2 | 14.3 | 16.7 | 18.0 | 16.5 |
| Clonidine | 13.9 | 17.8 | 17.5 | 16.4 | 16.9 |
| Haloperidol | 31.0 | 24.8 | 20.7 | 20.3 | 22.0 |
| Dexmedetomidine | 70.0 | 48.1 | 20.0 | 14.5 | 26.8 |
| Propofol | 84.4 | 2.2 | 5.2 | 4.8 | 9.0 |
| Valproic Acid | 4.1 | 2.9 | 3.2 | 2.8 | 3.0 |
| Carbamazepine | 0.3 | 0.4 | 0.4 | 0.6 | 0.5 |
| Ketamine | 7.7 | 1.2 | 0.8 | 0.6 | 1.2 |

| **No Delirium Tremens Medication Usage** | **ICU receiving IMV** | **ICU not receiving IMV** | **Intermediate** | **General Wards** | **Overall** |
| --- | --- | --- | --- | --- | --- |
| Days 1-5, n = | 1,860 | 14,303 | 35,815 | 136,711 | 188,689 |
| **Benzodiazepines** |  |  |  |  |  |
| Any Benzodiazepine | 95.0 | 96.1 | 95.0 | 89.7 | 91.2 |
| Lorazepam | 84.7 | 90.4 | 87.1 | 75.7 | 79.0 |
| Chlordiazepoxide | 24.2 | 37.1 | 34.4 | 33.1 | 33.5 |
| Diazepam | 18.3 | 20.1 | 18.3 | 15.8 | 16.6 |
| Midazolam | 53.3 | 4.3 | 2.4 | 1.4 | 2.4 |
| **Phenobarbital** | 21.4 | 13.8 | 10.4 | 8.6 | 9.5 |
| **Other Agents** |  |  |  |  |  |
| Any Other Agent | 94.6 | 49.3 | 39.3 | 41.8 | 42.4 |
| Gabapentin | 11.9 | 16.2 | 19.2 | 25.1 | 23.2 |
| Clonidine | 11.1 | 14.6 | 15.2 | 17.5 | 16.8 |
| Haloperidol | 17.3 | 11.7 | 6.9 | 4.9 | 5.9 |
| Dexmedetomidine | 56.1 | 21.6 | 3.7 | 1.3 | 3.9 |
| Propofol | 82.6 | 2.2 | 2.6 | 1.4 | 2.4 |
| Valproic Acid | 4.1 | 2.1 | 2.1 | 2.1 | 2.1 |
| Carbamazepine | 0.4 | 0.4 | 0.4 | 0.7 | 0.6 |
| Ketamine | 6.5 | 0.6 | 0.3 | 0.2 | 0.3 |

Top figure refers to patients with delirium tremens and the bottom figure to patients without delirium tremens. Dark green corresponds to medications with the highest use. Dark red corresponds to medications with the lowest use. The column headings correspond to patients’ level of care on the first day of hospitalization (i.e., at admission) whereas the percentages incorporate medication use over the first 5 days of admission.

**eFigure 3: Heatmaps of AWS medication use stratified by hospital case volume and level of care at admission**

| **High Volume Hospital Medication Usage** | **ICU receiving IMV** | **ICU not receiving IMV** | **Intermediate** | **General Wards** | **Overall** |
| --- | --- | --- | --- | --- | --- |
| Days 1-5, n = | 4,492 | 21,631 | 44,270 | 151,748 | 222,141 |
| **Benzodiazepines** |  |  |  |  |  |
| Any Benzodiazepine | 96.4 | 96.7 | 95.7 | 90.4 | 92.2 |
| Lorazepam | 88.7 | 92.0 | 88.5 | 77.4 | 81.3 |
| Chlordiazepoxide | 28.6 | 37.6 | 36.0 | 33.7 | 34.4 |
| Diazepam | 22.4 | 22.5 | 19.6 | 16.5 | 17.8 |
| Midazolam | 55.7 | 5.8 | 3.3 | 2.0 | 3.7 |
| **Phenobarbital** | 29.3 | 19.6 | 12.8 | 9.8 | 11.8 |
| **Other Agents** |  |  |  |  |  |
| Any Other Agent | 95.9 | 60.3 | 43.4 | 43.7 | 46.3 |
| Gabapentin | 12.4 | 15.2 | 18.6 | 24.5 | 22.2 |
| Clonidine | 12.6 | 15.3 | 15.9 | 17.5 | 16.8 |
| Haloperidol | 25.6 | 18.3 | 10.6 | 7.2 | 9.3 |
| Dexmedetomidine | 65.5 | 35.8 | 8.0 | 3.3 | 8.7 |
| Propofol | 83.8 | 2.3 | 3.3 | 1.9 | 3.8 |
| Valproic Acid | 4.5 | 2.6 | 2.4 | 2.2 | 2.3 |
| Carbamazepine | 0.3 | 0.4 | 0.4 | 0.7 | 0.6 |
| Ketamine | 7.3 | 1.0 | 0.4 | 0.2 | 0.5 |

| **Low Volume Hospital Medication Usage** | **ICU receiving IMV** | **ICU not receiving IMV** | **Intermediate** | **General Wards** | **Overall** |
| --- | --- | --- | --- | --- | --- |
| Days 1-5, n = | 691 | 5,747 | 4,727 | 11,965 | 23,130 |
| **Benzodiazepines** |  |  |  |  |  |
| Any Benzodiazepine | 98.8 | 98.1 | 96.3 | 93.6 | 95.4 |
| Lorazepam | 94.6 | 94.1 | 91.6 | 85.3 | 89.0 |
| Chlordiazepoxide | 36.6 | 44.6 | 40.3 | 38.7 | 40.5 |
| Diazepam | 22.6 | 21.2 | 14.6 | 17.1 | 17.8 |
| Midazolam | 61.4 | 4.4 | 3.0 | 2.5 | 4.8 |
| **Phenobarbital** | 21.6 | 11.7 | 7.2 | 7.1 | 8.7 |
| **Other Agents** |  |  |  |  |  |
| Any Other Agent | 97.0 | 57.5 | 43.7 | 40.3 | 47.0 |
| Gabapentin | 10.1 | 15.6 | 18.0 | 17.1 | 16.7 |
| Clonidine | 15.2 | 19.4 | 15.3 | 15.4 | 16.4 |
| Haloperidol | 29.4 | 16.7 | 10.6 | 9.4 | 12.1 |
| Dexmedetomidine | 61.8 | 28.4 | 8.9 | 5.7 | 13.7 |
| Propofol | 83.4 | 1.9 | 3.0 | 2.7 | 5.0 |
| Valproic Acid | 1.7 | 1.9 | 2.2 | 2.0 | 2.0 |
| Carbamazepine | 0.4 | 0.4 | 0.4 | 0.9 | 0.6 |
| Ketamine | 7.1 | 0.6 | 0.3 | 0.3 | 0.6 |

Top figure refers to patients admitted to low case volume hospitals (hospitals below the overall median case volume for AWS) and the bottom figure to patients admitted to high case volume hospitals (hospitals at or above the overall median case volume for AWS). Dark green corresponds to medications with the highest use. Dark red corresponds to medications with the lowest use. The column headings correspond to patients’ level of care on the first day of hospitalization (i.e., at admission) whereas the percentages incorporate medication use over the first 5 days of admission.

**eFigure 4: Medication Flow Diagram for patients initially admitted to Intermediate Care**


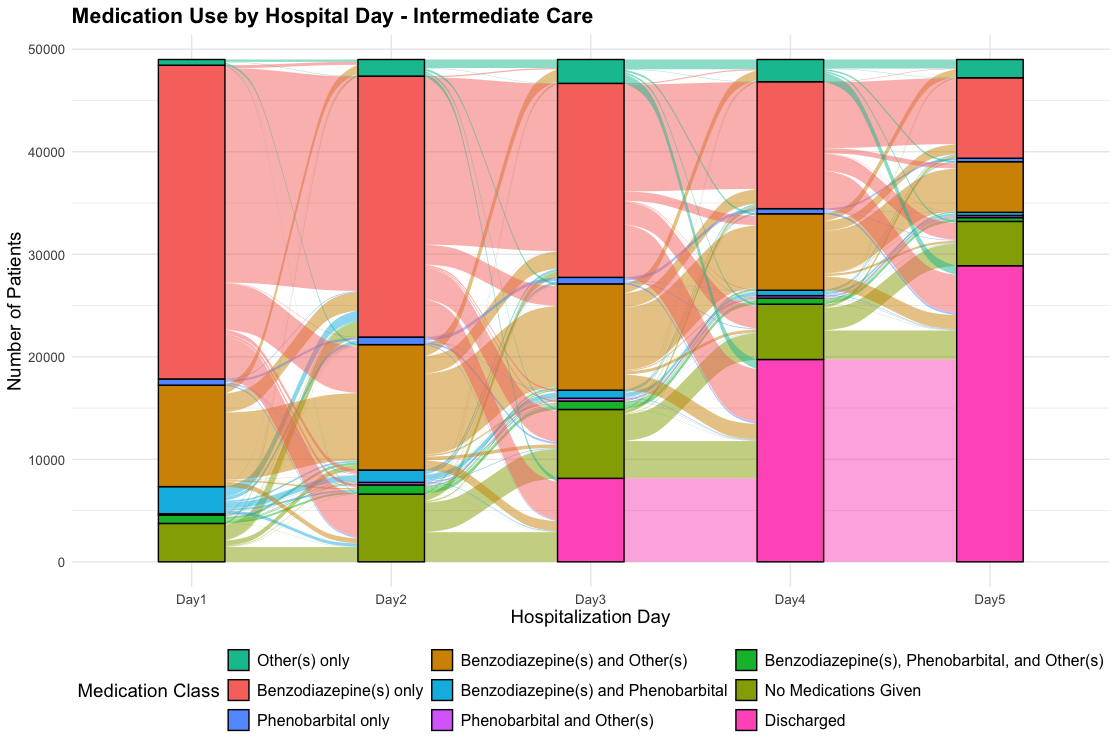


Alluvial flow diagram of hospital days 1 through 5 for patients admitted to intermediate care on hospital day 1.

**eFigure 5: Medication Flow Diagram for patients initially admitted to General Wards**


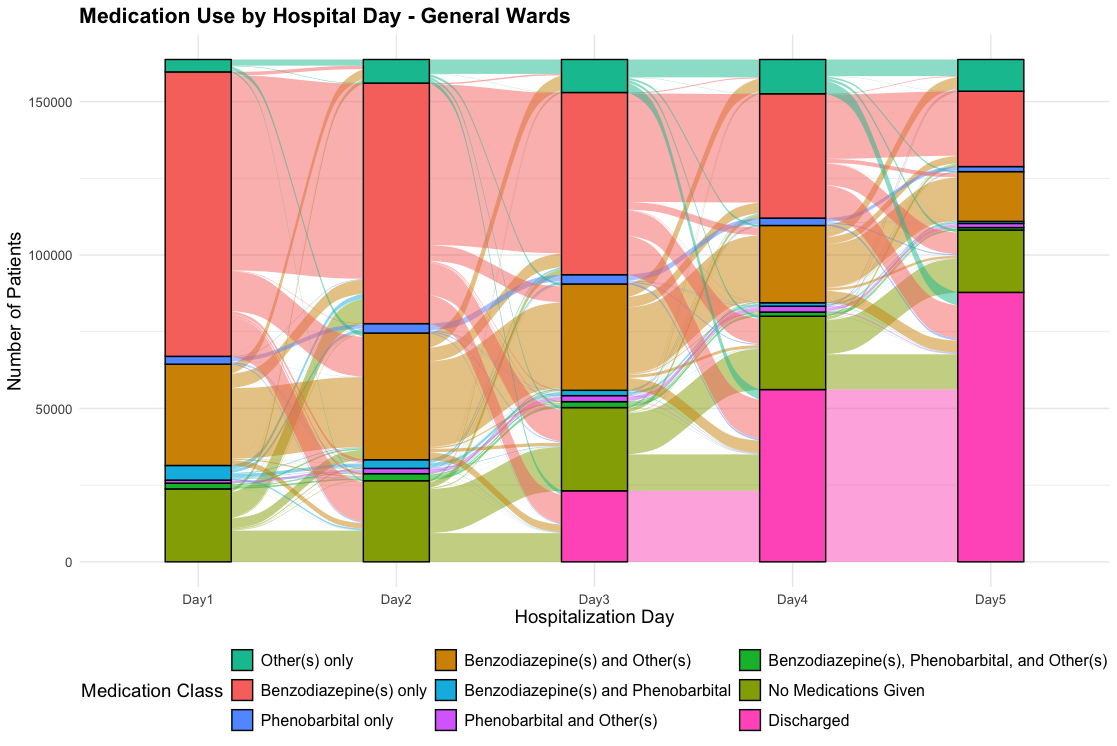


Alluvial flow diagram of hospital days 1 through 5 for patients admitted to the general wards on hospital day 1.

**eFigure 6: Medication Flow Diagram for patients initially admitted to ICUs and receiving IMV**


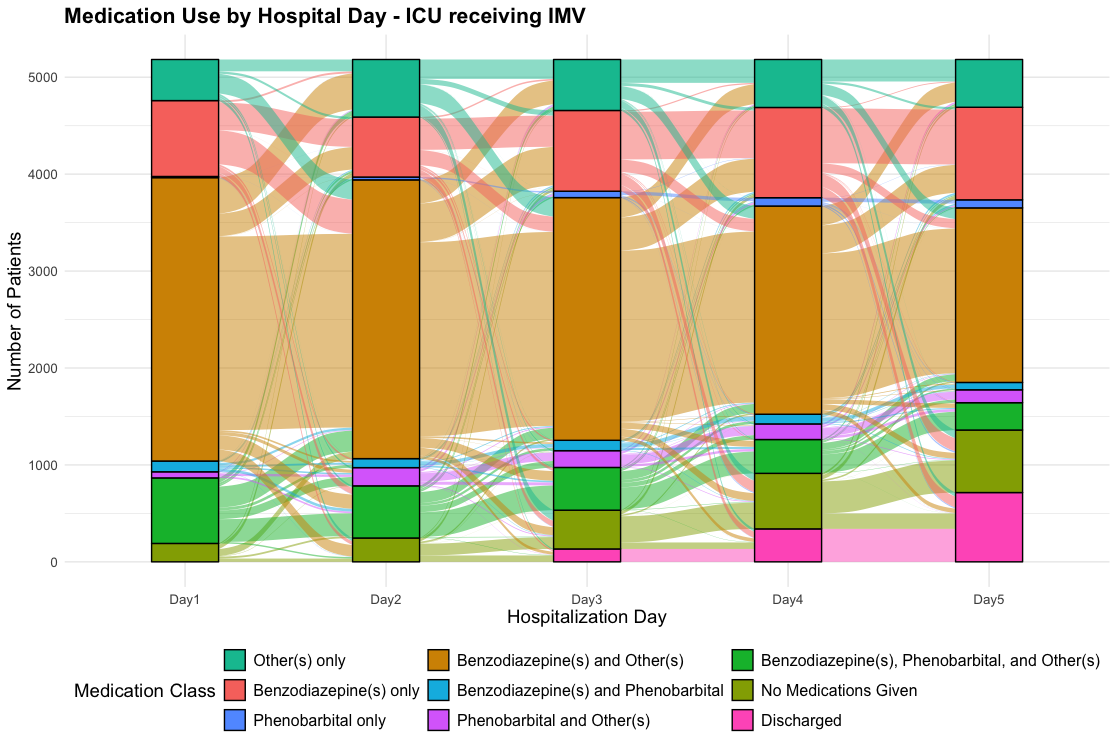


Alluvial flow diagram of hospital days 1 through 5 for patients admitted to the ICU on hospital day 1 and who received IMV during hospitalization.

**eFigure 7: Medication Flow Diagram for patients initially admitted to ICUs and not receiving IMV**


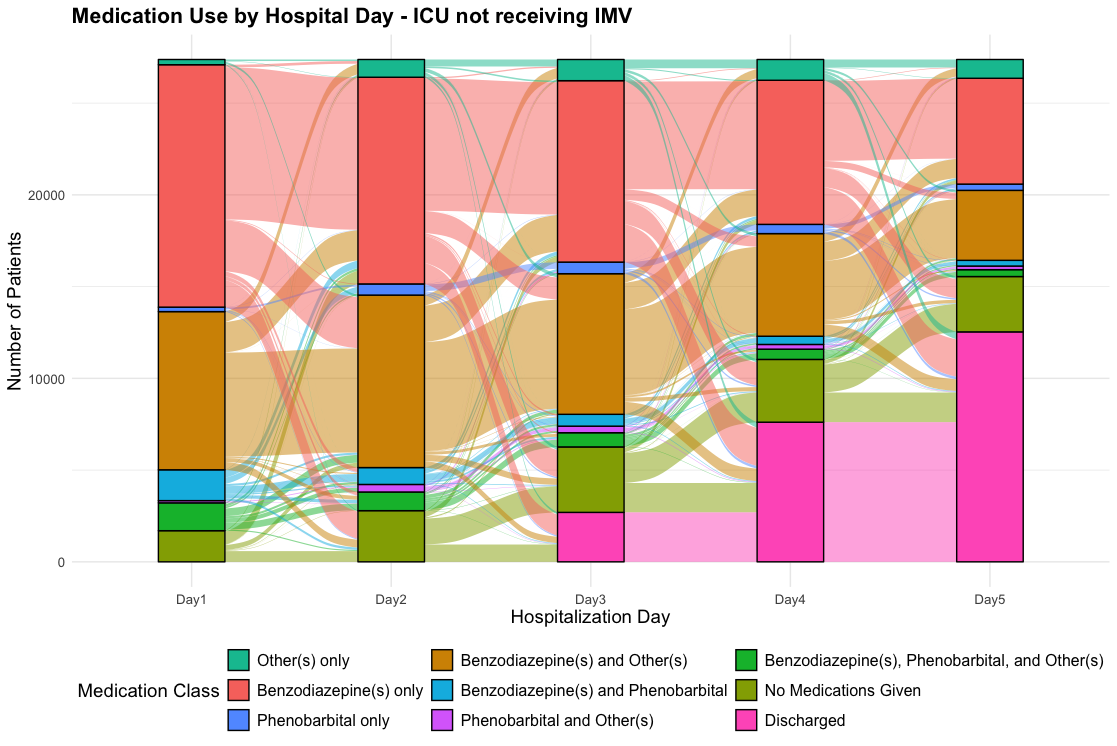


Alluvial flow diagram of hospital days 1 through 5 for patients admitted to the ICU on hospital day 1 and who did not receive IMV during hospitalization.

**eTable 2:** **Adjusted Odds Ratios (95% confidence intervals) for Benzodiazepine and Phenobarbital Administration**

| **Variable** | **Phenobarbital** | **Any Benzodiazepine** | **Lorazepam** | **Chlordiazepoxide** | **Diazepam** | **Midazolam** |
| --- | --- | --- | --- | --- | --- | --- |
| Age | 0.85 (0.84, 0.87) | 0.91 (0.89, 0.93) | 0.93 (0.91, 0.94) | 0.91 (0.90, 0.92) | 0.88 (0.87, 0.89) | 0.91 (0.89, 0.93) |
| Level of Care (versus General Ward) | | | | | | |
| ICU receiving IMV | 6.72 (6.19, 7.29) | 1.17 (0.97, 1.41) | 1.70 (1.54, 1.87) | 0.73 (0.68, 0.78) | 1.48 (1.37, 1.60) | 59.31 (55.21, 63.72) |
| ICU not receiving IMV | 3.87 (3.68, 4.07) | 2.11 (1.87, 2.37) | 2.66 (2.52, 2.81) | 1.15 (1.11, 1.19) | 1.64 (1.58, 1.71) | 2.69 (2.51, 2.88) |
| Intermediate Care | 1.49 (1.42, 1.56) | 1.93 (1.77, 2.11) | 1.89 (1.82, 1.97) | 1.14 (1.11, 1.18) | 1.23 (1.19, 1.28) | 1.47 (1.37, 1.58) |
| Gagne Comorbidity | 1.10 (1.08, 1.12) | 1.17 (1.14, 1.21) | 1.23 (1.22, 1.25) | 1.01 (1.00, 1.02) | 1.04 (1.02, 1.05) | 1.26 (1.23, 1.29) |
| Year (per one year increase) | 1.44 (1.43, 1.46) | 0.93 (0.93, 0.93) | 0.99 (0.98, 0.99) | 0.95 (0.95, 0.96) | 1.03 (1.03, 1.03) | 1.10 (1.10, 1.10) |
| Hospital size (versus 0-99) | | | | | | |
| 100-199 Beds | 0.91 (0.64, 1.30) | 1.17 (0.84, 1.64) | 1.03 (0.84, 1.28) | 1.21 (0.90, 1.63) | 0.92 (0.72, 1.16) | 1.11 (0.93, 1.33) |
| 200-299 Beds | 1.02 (0.71, 1.49) | 1.34 (0.94, 1.91) | 1.12 (0.89, 1.41) | 1.18 (0.85, 1.63) | 0.76 (0.59, 0.99) | 1.15 (0.95, 1.38) |
| 300-399 Beds | 1.01 (0.66, 1.54) | 1.00 (0.68, 1.46) | 1.02 (0.79, 1.32) | 1.04 (0.72, 1.51) | 0.82 (0.61, 1.09) | 1.16 (0.95, 1.42) |
| 400-499 Beds | 1.53 (0.93, 2.50) | 0.82 (0.53, 1.27) | 0.83 (0.61, 1.12) | 1.07 (0.69, 1.66) | 1.01 (0.72, 1.43) | 1.28 (1.02, 1.62) |
| 500+ Beds | 1.92 (1.24, 2.99) | 0.66 (0.44, 0.97) | 0.83 (0.63, 1.09) | 0.79 (0.53, 1.18) | 0.71 (0.52, 0.97) | 1.27 (1.03, 1.56) |
| Teaching Hospital | 1.48 (1.13, 1.95) | 0.91 (0.71, 1.16) | 0.93 (0.79, 1.11) | 0.81 (0.63, 1.04) | 1.15 (0.95, 1.40) | 1.20 (1.06, 1.35) |
| Urban Hospital | 0.85 (0.63, 1.15) | 0.81 (0.61, 1.08) | 0.96 (0.80, 1.16) | 0.92 (0.71, 1.20) | 0.90 (0.74, 1.11) | 0.88 (0.76, 1.02) |
| Safety Net Hospital | 0.85 (0.67, 1.13) | 0.83 (0.67, 1.05) | 0.75 (0.64, 0.87) | 1.01 (0.80, 1.27) | 1.19 (1.00, 1.42) | 1.05 (0.93, 1.18) |
| Location (versus East North Central) | | | | | | |
| East South Central | 0.45 (0.27, 0.74) | 0.86 (0.55, 1.33) | 0.87 (0.64, 1.17) | 1.66 (1.09, 2.54) | 1.06 (0.75, 1.48) | 1.06 (0.83, 1.35) |
| Middle Atlantic | 0.70 (0.47, 1.04) | 1.70 (1.19, 2.42) | 0.79 (0.62, 1.01) | 1.42 (0.99, 2.03) | 1.39 (1.05, 1.83) | 1.41 (1.18, 1.69) |
| Mountain | 1.10 (0.67, 1.80) | 0.96 (0.62, 1.48) | 0.69 (0.51, 0.94) | 1.36 (0.87, 2.12) | 2.03 (1.43, 2.87) | 1.19 (0.95, 1.49) |
| New England | 6.33 (3.12, 12.84) | 0.75 (0.41, 1.38) | 1.07 (0.67, 1.69) | 0.34 (0.17, 0.66) | 2.15 (1.29, 3.58) | 1.14 (0.82, 1.59) |
| Pacific | 1.99 (1.33, 2.97) | 1.13 (0.78, 1.63) | 1.12 (0.87, 1.44) | 1.84 (1.28, 2.64) | 1.08 (0.81, 1.44) | 1.14 (0.94, 1.39) |
| South Atlantic | 0.67 (0.47, 0.93) | 1.44 (1.06, 1.95) | 0.98 (0.79, 1.21) | 1.77 (1.31, 2.40) | 1.05 (0.83, 1.34) | 1.25 (1.06, 1.47) |
| West North Central | 0.22 (0.13, 0.39) | 1.21 (0.76, 1.94) | 0.82 (0.60, 1.13) | 0.95 (0.60, 1.50) | 0.81 (0.56, 1.16) | 0.81 (0.63, 1.04) |
| West South Central | 0.25 (0.16, 0.40) | 2.34 (1.54, 3.55) | 0.98 (0.75, 1.29) | 2.86 (1.95, 4.21) | 0.88 (0.65, 1.19) | 1.04 (0.84, 1.29) |
| Median Odds Ratio | 4.21 (4.03, 4.40) | 3.09 (2.96, 3.23) | 2.26 (2.33, 2.39) | 4.05 (3.94, 4.17) | 2.89 (2.81, 2.98) | 1.80 (1.76, 1.84) |

**eTable 3:** **Adjusted Odds Ratios (95% confidence intervals) for Other Agent Administration**

| **Variable** | **Any Other Agent** | **Gabapentin** | **Clonidine** | **Haloperidol** | **Dexmedetomidine** | **Propofol** | **Valproic Acid** | **Carbamazepine** | **Ketamine** |
| --- | --- | --- | --- | --- | --- | --- | --- | --- | --- |
| Age | 1.04 (1.03, 1.05) | 1.01 (0.99, 1.02) | 1.02 (1.01, 1.03) | 0.97 (0.95, 0.98) | 0.88 (0.87, 0.90) | 0.92 (0.89, 0.94) | 0.94 (0.91, 0.96) | 0.91 (0.86, 0.96) | 0.80 (0.76, 0.86) |
| Level of Care (versus General Ward) | | | | | | | | | |
| ICU receiving IMV | 61.45 (51.13, 73.85) | 0.47 (0.43, 0.51) | 0.89 (0.82, 0.97) | 3.97 (3.71, 4.25) | 61.47 (57.18, 66.07) | 242.93 (222.38, 265.37) | 1.91 (1.65, 2.21) | 0.58 (0.36, 0.95) | 29.55 (25.19, 34.67) |
| ICU not receiving IMV | 2.29 (2.22, 2.36) | 0.70 (0.67, 0.73) | 1.14 (1.09, 1.19) | 2.64 (2.53, 2.75) | 14.83 (14.20, 15.49) | 0.85 (0.78, 0.94) | 1.28 (1.17, 1.40) | 0.66 (0.54, 0.81) | 3.82 (3.20, 4.57) |
| Stepdown | 1.01 (0.98, 1.04) | 0.78 (0.75, 0.80) | 1.06 (1.02, 1.10) | 1.35 (1.29, 1.41) | 1.89 (1.80, 1.98) | 1.34 (1.25, 1.44) | 0.98 (0.91, 1.06) | 0.70 (0.59, 0.83) | 1.59 (1.32, 1.92) |
| Gagne Comorbidity | 1.18 (1.17, 1.19) | 1.12 (1.11, 1.13) | 0.94 (0.93, 0.95) | 1.15 (1.14, 1.17) | 1.38 (1.36, 1.41) | 1.51 (1.47, 1.54) | 1.06 (1.04, 1.09) | 0.99 (0.93, 1.05) | 1.13 (1.06, 1.19) |
| Year (per one year increase) | 1.02 (1.02, 1.02) | 1.09 (1.09, 1.09) | 0.96 (0.96, 0.97) | 0.95 (0.95, 0.95) | 1.12 (1.12, 1.12) | 1.01 (1.01, 1.01) | 1.02 (1.02, 1.02) | 0.85 (0.85, 0.85) | 1.14 (1.10, 1.18) |
| Hospital size (versus 0-99) | | | | | | | | | |
| 100-199 Beds | 1.04 (0.92, 1.18) | 1.01 (0.85, 1.20) | 0.88 (0.75, 1.03) | 1.00 (0.86, 1.15) | 1.72 (1.38, 2.15) | 1.12 (0.94, 1.35) | 1.02 (0.80, 1.28) | 0.93 (0.62, 1.38) | 0.98 (0.71, 1.37) |
| 200-299 Beds | 1.00 (0.88, 1.15) | 0.85 (0.70, 1.02) | 0.89 (0.75, 1.05) | 0.92 (0.79, 1.07) | 1.54 (1.22, 1.95) | 1.17 (0.97, 1.41) | 1.13 (0.89, 1.44) | 0.87 (0.58, 1.30) | 0.76 (0.54, 1.08) |
| 300-399 Beds | 1.07 (0.91, 1.24) | 0.95 (0.77, 1.17) | 0.89 (0.73, 1.08) | 1.03 (0.87, 1.22) | 1.65 (1.27, 2.15) | 1.10 (0.90, 1.34) | 1.36 (1.05, 1.76) | 1.09 (0.71, 1.67) | 0.97 (0.68, 1.38) |
| 400-499 Beds | 1.03 (0.86, 1.23) | 0.94 (0.73, 1.20) | 0.92 (0.73, 1.15) | 1.03 (0.85, 1.25) | 1.34 (0.98, 1.83) | 1.28 (1.02, 1.60) | 1.36 (1.01, 1.82) | 0.82 (0.50, 1.32) | 1.12 (0.76, 1.64) |
| 500+ Beds | 1.12 (0.95, 1.32) | 1.12 (0.90, 1.40) | 0.87 (0.71, 1.07) | 0.94 (0.79, 1.12) | 1.39 (1.05, 1.83) | 1.14 (0.93, 1.40) | 1.04 (0.79, 1.36) | 0.80 (0.52, 1.25) | 1.10 (0.77, 1.56) |
| Teaching Hospital | 0.88 (0.79, 0.97) | 1.07 (0.93, 1.23) | 0.72 (0.63, 0.82) | 0.91 (0.82, 1.02) | 0.92 (0.77, 1.09) | 0.96 (0.85, 1.08) | 1.03 (0.88, 1.21) | 0.89 (0.70, 1.15) | 1.24 (1.01, 1.52) |
| Urban Hospital | 1.22 (1.09, 1.36) | 1.17 (1.00, 1.36) | 1.15 (1.00, 1.32) | 1.09 (0.96, 1.23) | 1.30 (1.08, 1.58) | 1.08 (0.93, 1.26) | 1.05 (0.86, 1.28) | 1.16 (0.83, 1.61) | 0.92 (0.71, 1.20) |
| Safety Net Hospital | 0.98 (0.89, 1.08) | 0.93 (0.82, 1.06) | 1.02 (0.91, 1.15) | 0.99 (0.89, 1.09) | 0.69 (0.59, 0.82) | 0.90 (0.80, 1.01) | 1.07 (0.93, 1.25) | 1.51 (1.20, 1.90) | 1.04 (0.86, 1.26) |
| Location (versus East North Central) | | | | | | | | | |
| East South Central | 0.98 (0.81, 1.17) | 0.59 (0.46, 0.75) | 1.65 (1.32, 2.07) | 1.07 (0.87, 1.30) | 0.66 (0.48, 0.90) | 1.35 (1.07, 1.69) | 0.87 (0.64, 1.18) | 0.65 (0.38, 1.10) | 0.87 (0.56, 1.34) |
| Middle Atlantic | 0.73 (0.63, 0.84) | 0.71 (0.58, 0.87) | 0.73 (0.60, 0.88) | 1.05 (0.90, 1.23) | 0.55 (0.43, 0.71) | 0.92 (0.78, 1.10) | 0.90 (0.72, 1.13) | 0.74 (0.51, 1.07) | 1.00 (0.74, 1.35) |
| Mountain | 0.88 (0.74, 1.06) | 0.78 (0.60, 1.00) | 0.68 (0.54, 0.87) | 1.10 (0.90, 1.34) | 1.08 (0.79, 1.47) | 1.21 (0.98, 1.50) | 0.87 (0.65, 1.16) | 0.77 (0.47, 1.25) | 1.15 (0.81, 1.65) |
| New England | 1.58 (1.21, 2.07) | 1.79 (1.24, 2.57) | 1.30 (0.93, 1.83) | 1.52 (1.15, 2.02) | 0.62 (0.39, 0.98) | 1.17 (0.87, 1.59) | 2.13 (1.45, 3.12) | 0.86 (0.45, 1.63) | 1.08 (0.66, 1.79) |
| Pacific | 1.14 (0.98, 1.32) | 1.17 (0.95, 1.43) | 1.29 (1.07, 1.57) | 0.92 (0.78, 1.09) | 1.19 (0.93, 1.54) | 0.91 (0.75, 1.09) | 1.11 (0.87, 1.41) | 0.62 (0.41, 0.93) | 1.21 (0.87, 1.66) |
| South Atlantic | 1.01 (0.89, 1.14) | 0.65 (0.55, 0.77) | 1.35 (1.15, 1.59) | 1.36 (1.19, 1.56) | 0.85 (0.69, 1.06) | 0.93 (0.80, 1.09) | 0.86 (0.70, 1.05) | 1.14 (0.83, 1.57) | 1.22 (0.94, 1.58) |
| West North Central | 0.90 (0.74, 1.09) | 0.79 (0.61, 1.02) | 1.09 (0.85, 1.39) | 1.31 (1.07, 1.61) | 0.72 (0.52, 1.00) | 0.90 (0.71, 1.15) | 0.56 (0.40, 0.77) | 0.98 (0.61, 1.60) | 0.92 (0.62, 1.36) |
| West South Central | 0.81 (0.69, 0.96) | 0.65 (0.52, 0.82) | 0.90 (0.73, 1.11) | 1.07 (0.90, 1.28) | 0.61 (0.46, 0.80) | 0.94 (0.77, 1.15) | 0.54 (0.41, 0.72) | 1.05 (0.69, 1.59) | 0.89 (0.62, 1.28) |
| Median Odd Ratio | 1.65 (1.62, 1.67) | 2.00 (1.95, 2.04) | 1.99 (1.96, 2.04) | 1.75 (1.72, 1.78) | 2.54 (2.47, 2.61) | 1.69 (1.66, 1.74) | 2.03 (1.97, 2.09) | 2.60 (2.50, 2.71) | 1.91 (1.86, 1.97) |

**eFigure 8: Forest Plot of Adjusted Odds Ratios by Hospital Location**


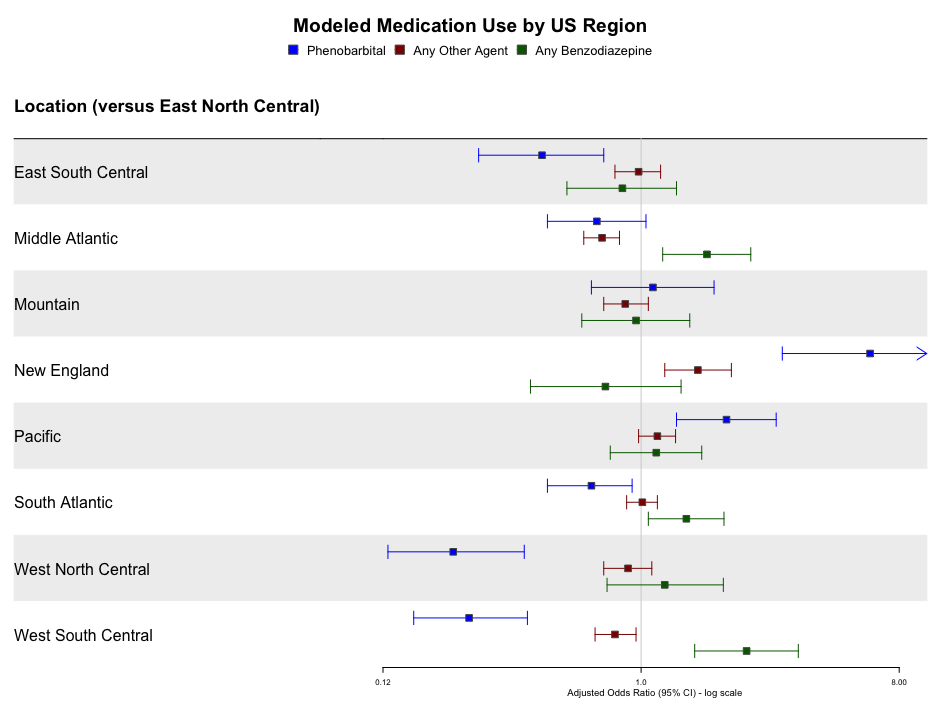


Adjusted odds ratios for each medication category presented as a forest plot. The 95% confidence interval x-axis logarithmic scale is from 0.12 to 8.00, with the New England phenobarbital adjusted odds ratio extending beyond the scale maximum to 12.84.

**eReferences**

1. Surgery Flags for Services and Procedures. Accessed February 16, 2024. https://hcup-us.ahrq.gov/toolssoftware/surgeryflags_svcproc/surgeryflagssvc_proc.jsp

2. Gagne JJ, Glynn RJ, Avorn J, Levin R, Schneeweiss S. A combined comorbidity score predicted mortality in elderly patients better than existing scores. *J Clin Epidemiol*. 2011;64(7):749-759. doi:10.1016/j.jclinepi.2010.10.004
